# Remote Physiotherapy for Children with ASD during the COVID-19 Pandemic: A Thematic Analysis of Physical Therapists’ Perspectives

**DOI:** 10.3390/jcm13061610

**Published:** 2024-03-11

**Authors:** Yael Harel, Alberto Romano, Meir Lotan

**Affiliations:** 1 “OTI”, Israeli Association for Autism, Gush Etsion 13, Givat Shmuel 54030, Israel; 2 Health System Management Department, Ariel University, Ariel 40700, Israel; 3 IRCCS Fondazione Don Carlo Gnocchi, 20148 Milan, Italy; 4 Physical Therapy Department, Health Science Faculty, Ariel University, Ariel 40700, Israel

**Keywords:** autism spectrum disorder, telerehabilitation, physical therapy modalities, physical therapy specialty

## Abstract

**Background**: Physical therapy plays a crucial role in addressing the physical challenges faced by individuals with autism spectrum disorder (ASD). Amidst the COVID-19 pandemic lockdown, physical therapists (PTs) working in special education centers for ASD children were tasked with deploying remote telehealth interventions (RTIs), an uncommon approach in physical therapy until then. The present article aims to describe and discuss the PTs’ perspective of using RTI with children with ASD during the national Israeli COVID-19 lockdown. **Methods**: Reports from 13 experienced PTs who treated and supported 244 children with ASD using RTIs over six weeks were analyzed. The study employed quantitative research methods, including freely written reports and discussions addressing the question “what were your experiences as a PT treating ASD children remotely during the nationwide COVID-19 lockdown?” **Results**: the reports were categorized into four main themes: (a) the implications of RTIs on the children; (b) the implications of RTIs on the PTs; (c) modifications for applying RTI; and (d) PTs’ family rapport as a necessary basis for RTI. Noteworthy findings include the unaffected implementation of RTIs by ASD severity level and the dependence of RTI’s success on parental availability and the ability of parents to tailor activities for their child. **Conclusions**: The findings of the current research suggest that PT services through RTIs are well-suited for individuals with ASD and their families.

## 1. Introduction

Autism spectrum disorder (ASD) is a neurodevelopmental disorder that encompasses a spectrum of symptoms and severity levels ranging from mild to severe. It is the most common pediatric diagnosis, with 36,500 new cases per year in the United States, a total of 730,000 cases across the United States [1], and an incidence rate of 1.75% in children [2]. Furthermore, research conducted in Israel indicated a rise in the prevalence of ASD among 8-year-old children, from 0.65% in 2015 [3] to 1.3% in 2018 [4] and 1.56% in 2021 [5]. ASD is characterized by impaired social reciprocity and communication and restricted, repetitive behavior, interests, and activities [6]. Compelling evidence from recent years suggests that many children with ASD present motor impairments, including in terms of basic and advanced developmental milestones such as crawling, walking, jumping, writing, using social gestures, and imitation [7,8,9,10,11]. In fact, abnormalities in motor coordination, posture, and gait, as well as poor muscle tone, were mentioned by both Kanner and Asperger in their original articles [12]. These symptoms appear to be neurologically and clinically significant elements of ASD [13] and are suspected to contribute to the manifestation of ASD core symptoms [14]. Children with ASD often present significant impairments in ADL abilities compared with age-matched controls [15,16]. These children demonstrate a higher dependency on the help of adults compared with typically developing children [17]. Indeed, such impairments can affect their independence, social engagement, and adaptive functioning, namely, the children and their families’ quality of life [18].

Therefore, one of the areas of focus for physical therapists (PTs) treating children with ASD is activities of daily living (ADL). ADL abilities are a significant part of adaptive behavior and are necessary for independent functioning in social situations, communication, daily living, and motor activities [19].

Physical therapy services are traditionally implemented through direct therapy (hands on), and remote PT services have typically been scarce. Until the outbreak of COVID-19, the vast majority of PT services relied heavily on nonresident visiting professionals [20] and on the patients’ capability to travel long distances to access services [21,22]. In the last decade, remote telehealth interventions (RTIs) have emerged as a means of providing greater access to allied health services, including PT services [23], as a way to shorten waiting lists, save costs, and enhance the availability of services to distant areas [22,24,25]. In pediatric care, the possibility of using PT services in the context of RTIs is considered a novel approach [26,27]. RTIs involve using technology for communication between patients and their healthcare providers [28,29]. A study that analyzed interviews with 307 Israeli healthcare professionals revealed that the sector that least used RTIs with pediatric patients pre-COVID-19 was the PTs sector. Only 13.13% of the 90 PTs participating in this study used RTIs as part of their provided services, and these were delivered at very low intensity, mainly through phone calls and by exchanging information with the parents [30]. We can, therefore, say that the use by pediatric PTs of RTIs with children with ASD is an undeveloped area.

During the first nationwide lockdown in Israel (14 March–19 April 2020), all educational facilities were closed, and all PT services were implemented only through remote technologies. OTI, the Israeli Association of Autism (formally the Association for Children at Risk), is responsible for providing allied health services for hundreds of children with ASD in special educational facilities. During lockdown, these services were supplied in the form of RTIs, aiming to maintain the interaction, preserve the intervention process, and ease the long stay at home for both the children and their families. Different approaches to RTI were implemented to adapt the treatments to the needs of children with ASD and their families. Some children were able to participate in online PT sessions, mostly with parental mediation, while others were led by their parents through short video clips or lists of exercises with therapeutic goals provided by the child’s PT.

Despite the rehabilitation professional’s dedication and rapidity in embracing communication technologies as a tool for providing their services, RTI was, and still is, a newborn approach [26,31]. Despite the growing interest and body of existing literature in the field of telerehabilitation and RTIs, PTs’ perspective in implementing them with children with developmental disabilities such as ASD remains scarce. Investigating how PTs perceive RITs, their benefits and challenges, and the aspects that influence their readiness and effectiveness in using them is essential to designing and evaluating new effective interventions to promote and support telerehabilitation practice. As part of the current study, an attempt was made to understand the participants’ point of view, to unfold their experiences and their meaning prior to scientific explanation [32], and to draw clinical insights based on those experiences. Therefore, the aim of the present article is to describe and discuss PTs’ perspective of using telehealth to deliver assessments and interventions with children with ASD during the national Israeli COVID-19 lockdown, with particular emphasis on how this compares with face-to-face service delivery, how PTs have adapted their interventions for working with children with ASD remotely, and how the children’s families were involved into the RTI development.

## 2. Materials and Methods

The current study was conducted based on the principles of phenomenological qualitative research, which allows the researcher to capture the feelings and thoughts of several individuals about a phenomenon they have in common [32,33].

### 2.1. Ethics

The Ariel University IRB (AU-HEA-ML-20221011) and the OTI research committee approved the present research. All participants agreed to participate by supplying their comments after receiving an explanation regarding the involved research.

### 2.2. Participants

An open invitation to participate in the current research was sent via e-mail to all physical therapists working within the OTI association. Responses from a total of 13 PTs (mean age in years: 41.4 ± 7.6) with experience treating children with ASD (mean years of experience: 5.8 ± 2.9) were received (see Table 1). All participating PTs worked for the OTI association and treated, in total, 250 children aged 2–7 with all severity levels of ASD.

### 2.3. Procedure

Participating PTs were invited to elaborate freely, in writing, on their thoughts and experiences providing RTI services to children with ASD during lockdown. The instructions were deliberately general to collect the most authentic responses and minimize external influences. All participants replied within two weeks of the end of lockdown (second half of April 2020). 

### 2.4. Data Analysis 

Thematic analysis was used to analyze the qualitative data based on the six-step method, providing a structured approach to conducting thematic analysis in qualitative research:

- Familiarization with the data: in this step, researchers become acquainted with the data and participants’ experiences by reading and rereading it to gain a deep understanding of its content and contexts. Notes were collected on initial impressions, patterns, and recurring ideas.
- Data coding: the data are systematically coded by assigning labels or tags to data segments relevant to the research question. Codes can be inductive (emerging from the data) or deductive (based on existing theories). These initial codes are often descriptive and capture the essence of the data.
- Searching for themes: researchers identify patterns or commonalities across the coded data by looking for similarities, differences, and recurring ideas. Potential themes emerge by grouping related codes and finding patterns or connections within the data.
- Theme review: this step involves reviewing and refining the identified themes to ensure they accurately represent the data. Identified themes are discussed within the research team to consider alternative interpretations and ensure that themes resonate with the data.
- Defining and naming themes: each theme is defined, clearly articulated, and labeled based on the content and meaning extracted from the data. Naming themes is crucial, as it helps to capture the essence of each identified pattern or concept succinctly.
- Producing the final report: the final step involves writing up the findings of the thematic analysis. Researchers present the identified themes, supported by quotes or examples from the data, to provide a rich and detailed account of the patterns observed. The report should include a discussion of findings, limitations, and recommendations that emerged from the data [34].

By following these key steps in thematic analysis, researchers can systematically analyze qualitative data in rehabilitation research, uncovering meaningful insights and contributing to a deeper understanding of individuals’ experiences and perspectives in the context of rehabilitation programs. In addition, to obtain evidence to corroborate the accuracy of current results, triangulation was applied. This method enhances the trustworthiness of the interpretation, reduces the possibility of bias, and leads to richer data [35]. The triangulation in the current study was achieved by contacting the participants after the initial data collection and preliminary analysis to discuss their observations further and confirm the researchers’ understanding of specific comments. Then, the newly added information, comments, and clarifications were added to the text analyzed through the thematic analysis. 

## 3. Results

Based on the six-step thematic analysis method, four key themes were identified and then underpinned by subthemes (see Table 2); examples of participants’ quotes are presented below to illustrate each subtheme.

### 3.1. Theme One: The Implications of RTI on Children with ASD

#### 3.1.1. The Effect of Staying at Home on Children’s Behavior

Some PTs noticed that children returned to kindergarten more relaxed and regulated after the extended stay at home with their parents:

*“Many children came back calmer and more sensory-regulated after the long period of staying at home with their parents.”*

(PT #8)

Children even demonstrated behavioral and functional improvement, which manifested mainly as enhanced ability to communicate, decreased pervasive behaviors, and higher self-confidence:

*“I noticed a decrease in stereotypical behaviors and self-stimulation up to the extinction of these types of behaviors. I felt that they gained higher self-confidence and were more aware of occurrences in the surrounding environment.”*

(PT #11)

#### 3.1.2. The Relation between ASD Severity and the Ability to Receive RTI Services

Participating PTs were in significant agreement that children’s functional levels do not predict their ability to participate in RTI sessions. Moreover, for some (high-functioning) children, the excitement of seeing the therapist at home on the screen was actually counterproductive to the RTI interaction:

*“Surprisingly, I did not find any relation between the children’s ASD level and their ability to enjoy remote rehabilitation services.”*

(PT #1)

*“Children considered to have low functional and communicative abilities were able to maintain a remote session, while other “high-functioning” children totally avoided the situation and withdrew from any connection with the kindergarten team members.”*

(PT # 8)

*“Some parents of “high-functioning” children reported extremely excited behavior in reaction to the online communication with the therapist that made it difficult to calm them down for a long time after the session.”*

(PT #12)

#### 3.1.3. The Child–PT Bond during RTI 

Children who attended the RTI sessions did not necessarily sit in front of the computer. Many needed to move around in order to participate and cooperate with the PT:

*“Many children needed to move and found it difficult to keep a static position in front of the computer for more than a few seconds. However, the fact that they did not stay seated in front of the computer did not mean they were not attentive to the therapist.”*

(PT #1)

This insight suggests that the bond between the PT involved in an RTI and the child is kept despite the fact the child is mobile and did not stay seated in front of the screen for the whole session.

RTI sessions helped maintain the connection between the child and the PT and made the return to kindergarten easier and more effortless in terms of renewing the relationship.

*“I felt that the sustained communication with the child contributed to a smoother return to the kindergarten and helped to maintain the therapeutic relationship.”*

(PT #13)

### 3.2. Theme Two: The Implications of RTI on the PT

#### 3.2.1. Preintervention Requirements for Applying RTI

Some PTs emphasized the importance of preparing familiar toys in advance as a way of promoting the child’s cooperation:

*“PTs promoted better and faster communication with the child through RTI when they prepared favorite games or objects they knew the child was attached to following a conversation with the parents, in advance.”*

(PT #1)

Flexibility seemed to be an essential attribute of RTI with children with ASD:

*“The PT needs to demonstrate flexible thinking and rapidly adjust the goals and attitude to the RTI model […] Working with the RTI model compelled the PTs to be flexible with their working hours in order to address the families’ needs.”*

(PT #2)

#### 3.2.2. The Impact of RTI on the PT’s Personal Life

Some PTs felt uncomfortable with the exposure and the penetration of their private life. They felt fragile and expected a better understanding of their needs:

*“Some of the PT’s reported that they felt vulnerable and unprotected by the expectation of filming themselves, exposed in their homes, of being available and supporting others, while at the same time taking care of their own lives and families during the lockdown.”*

(PT #8)

Many PTs commented on RTI as a time-consuming service:

*“It is necessary to maintain a permanent schedule. When a family did not respond to my call at the set hour, I sent a message saying I remained available for them for an additional half an hour. Most of the time, it encouraged the family to answer.”*

(PT #8)

There were other challenges as well. First, PTs had to cope with the need to learn and use new technologies rapidly:

*“It took me time to get a grip on all the new technologies, which forced me to spend much more time than I was supposed to invest in the RTI sessions themselves.”*

(PT#8)

Second, they were required to work from home and around the clock while continuing to care for their own families:

*“It was quite challenging to continue working from home with small children all around, having no help, and trying to balance between working at nights (e.g., editing video clips, having team meetings, etc.) and maintaining my family routine.”*

(PT#10)

#### 3.2.3. The Impact of RTI on the PT’s Professional Experience

Opposing feelings were reported. Some reported feelings of frustration as their professional habits were disrupted:

*“Indirect treatment made it difficult to adjust the therapy to the child’s needs as we regularly do, especially when it was done by sending parents video clips to watch and apply with their children. Frequently, it raised feelings of frustration, affected the sense of creativity, and disrupted the confidence of helping the child improve.”*

(PT #4)

*“Some PTs expressed their concern that the PTs RTI sessions were more about maintaining the connection with the child and supporting the parents’ emotional status than working with the child on his or her motor skills.”*

(PT #8)

Other PTs noticed that within a short time, their creativity and interventional ideas had expanded:

*“I soon realized that, without any doubt, we will come out at the end of this period more creative, with a large array of ideas.”*

(PT #8)

*“Working from my home without any appropriate therapeutic equipment made me improvise and use household appliances as therapeutic elements.”*

(PT #10)

*“After a week or two of working online, thinking it would be impossible for me, I found out I was quite good and realized that I had some hidden skills within me.”*

(PT #12)

#### 3.2.4. The Role of Teamwork during RTI

The reporting PTs agreed that a positive relationship among team members and coordinative teamwork highly supported the therapists and the families during the lockdown and helped them provide appropriate RTI services:

*“Optimal teamwork was found to play a leading role during lockdown in terms of the therapists’ well-being and their continued ability to support the families and the children. This refers to coordinated communication, sharing families’ reactions and comments, making mutual decisions, supporting each other in stressful periods and situations, and more.”*

(PT #6)

*“Some teams nominated a case manager from the team who was responsible for keeping in touch with families that were finding it hard to stay connected at this point in time, sending them therapeutic materials collected from the child’s various therapists, and coordinating between the families and the team members.”*

(PT #7)

*“Many teams set up daily mini-meetings to get updated on the therapeutic process and responsiveness of the child and his or her family.”*

(PT #8)

### 3.3. Theme Three: Therapeutic Modifications for Applying RTI 

#### 3.3.1. Using RTI to Collect Information on the Child

One of the PTs added a critical comment on the advantage of RTI in providing a live perspective of the child in his or her natural home environment:

*“Entering the child’s home and family dynamics helped me gather more information about the child, which sometimes contributed possible explanations for the child’s behavior.”*

(PT #3)

#### 3.3.2. Setting Therapeutic Goals for RTI 

Several PTs emphasized the fact that RTIs have different goals than live interventions:

*“The goals of RTI are different than those set for work done within the (PT) therapy room.”*

(PT #5)

*“The goals were rephrased to meet the conditions of RTI sessions.”*

(PT #7)

#### 3.3.3. Therapeutic Principles of RTI

1. Preintervention considerations and adaptations—some PTs commented on the substantial effect of modulating the intervention according to the child’s home and family conditions:

*“Creativity is needed in adapting the therapeutic session to the home situation.”*

(PT #2)

*“The opportunity to “enter” the children’s houses allowed the PTs to adjust the RTI activities to the equipment available in each child’s home and even to invite siblings to take part in the sessions.”*

(PT #3)

*“Conducting RTI sessions became highly challenging with families who do not use computers or smartphones. However, creative ideas, good relationships with parents, and willingness on both sides sometimes helped conduct the RTI despite such challenges.”*

(PT #7)

2. The role of routine in RTI—the participating PTs emphasized the effect of maintaining a set routine, familiar activities, settings, and ongoing repetitions:

*“Maintaining a routine for children with ASD is a basic therapeutic principle. It is important to keep in mind and explain to the parents that repetitions are part of the therapeutic process. Variability in the session’s activities is expected when the child is ready for changes.”*

(PT #1)

*“Many children with ASD depend on repetitions and consistency in therapeutic sessions to cope with their autistic needs […] Repetitions in RTI sessions helped children remain in front of the computer longer and cooperate with the PT. Using familiar movement songs, maintaining the same activities with only minor variations from session to session created a familiar framework for the children and an experience they can look forward to.”*

(PT #2)

3. Practical recommendations—the participating PTs mentioned that the same therapeutic principles known from hands-on intervention were used in RTI sessions to encourage compliance, cooperation, and long-term communication.
  - Identify the topic of interest and raise the child’s motivation:
    *“Occasionally, exactly like when working in the therapy room, the success of the therapeutic session depends upon understanding what the child is interested in so you can include it into the activity, making it more interesting for the child.”*
    (PT #1)
  - Building relationships during RTI:
    *“Reflecting the situation for the child, as well as his or her reactions and feelings, helped establish good and trustworthy rapport with the child through RTI.”*
    (PT #1)
  - Music, songs, and stories:
    *“Music and songs helped tremendously in recruiting the children’s compliance and cooperation. It is important to use these tools while telling social stories, encouraging movement, teaching new motor skills, playing together, etc.”*
    (PT #1)
  - Familiar and consistent signals:
    *“Using familiar and consistent signals, such as acceptable rhythm or movement, helped maintain the child’s attention.”*
    (PT #1)
    *“Holiday songs motivated the children I worked with the most. We gained longer cooperation while strengthening their core muscles.”*
    (PT #4)
  - Gradual and continuous process:
    *“The therapeutic process through RTI must be gradual and continuous. Each session must refer to the previous one.”*
    (PT #6)

#### 3.3.4. Parental Involvement

The participating PTs were in consensus regarding the fundamental role of parents in the success of the RTI:

*“It is highly recommended to encourage parents to take an active part in the session. I noticed that it helped strengthen the parent-child connection.”*

(PT #1)

*“Parents’ recruitment and involvement is essential to ensure RTI success, starting with preparing the child, handling the technical settings, and cooperating with the PT during the remote intervention session.”*

(PT #3)

*“During lockdown, I had the chance to experience the effectiveness of treating a low-functioning child by instructing the parents. I must say that the child benefited more, even with the direct therapy session with me in the therapy room.”*

(PT #3)

*“The remote sessions significantly improved my relationships with most of the parents. The common attunement to each child enabled the assembly of a complete picture of the child in various places and settings.”*

(PTs #7,11)

*“The parents were found as the most significant factor in the success or failure of the RTI […] with proper moderation, even children who we considered unable to be involved in such a session, were able to enjoy and participate in the session.”*

(PTs #3,8)

### 3.4. Theme Four: Therapist-Parent Rapport as a Necessary Basis for RTI

#### 3.4.1. Dynamics of the Relationship

Most of the PTs emphasized the importance of coordinating expectations with parents prior to the beginning of RTI with their child:

*“It is crucial to talk directly with the parents and create a new therapeutic contract when meeting the child and parents within the home setting.”*

(PT #2)

*“To benefit from the RTI, the parents must trust the therapist, maintaining an open and continuous dialog between the two sides.”*

(PT #3)

*“Using RTI, the communication with the parents changed from receiving reports from the therapeutic sessions regarding their child’s therapeutic process and progression to consultations on challenges they faced and ways of coping with them.”*

(PT #10)

#### 3.4.2. Perceptions of the Child’s Functional Abilities 

Some of the PTs found RTI to be an opportunity to understand both the differences in the way parents and therapists view the child’s functioning and what parents cope with when raising a child with ASD:

*“Through the intensive interaction with parents, I realized how wide the gap can be between the therapists’ and the parents’ perception of the child’s functioning. In addition, my perspective on the difficulties families of children with ASD face became more authentic and precise.”*

(PT #6)

*“In some cases, the RTI sessions were an excellent opportunity to expose the parents to their child’s motor abilities/challenges.”*

(PT #8)

## 4. Discussion

In this paper, the PTs’ perspective on RTI provision to children with ASD and their families was analyzed qualitatively. RTI services were delivered through different modalities in accordance with child and family availability, namely, phone calls, video calls, online platforms, video clips sent to the families, exercise booklets, and other materials available for remote contact [36]. The information elicited from the interviews referred to the following four main themes:

- Theme One: the implications of RTI for children with ASD;
- Theme Two: the implications of RTI for the PT;
- Theme Three: therapeutic modifications for applying RTI;
- Theme Four: PT–family rapport as a necessary basis for RTI.

The results are discussed through the lens of a review of the literature and relevant theories.

### 4.1. Theme One: The Implications of RTI on Children with ASD 

#### 4.1.1. The Effect of Staying at Home on Children’s Behavior 

Based on the participating PTs’ insights, it is our impression that staying at home for a limited period had a more positive than negative effect on the behavior of children with ASD. This was manifested by more regulated and calmer reactions, less stereotypical behavior, and better communication between the children and their kindergarten teams upon returning to kindergarten when the lockdown was lifted. Similar results (whereby most children improved and only a minority regressed) were reported by others [37,38]; according to these studies, among those children receiving RTI, 85% experienced benefits from this type of intervention. 

#### 4.1.2. The Relation between ASD Severity Level and the Ability to Receive RTI Services 

Our findings are in line with and expand a previous study (also regarding telehealth intervention) that claimed that the child’s initial ASD severity level does not predict his or her response to such an intervention [39]. We noticed that high-functioning children frequently demonstrated difficulties in adjusting to new settings and cooperating, while children with lower abilities reacted much better. We realized that the child’s response to RTI depends mainly on parental involvement, as elaborated in Theme Three. 

These findings do not agree with other reports that suggest that ASD severity might negatively influence clinical intervention outcomes [40,41,42,43]. The intervention programs mentioned above were all performed within “hands-on” situations, thereby enhancing the unicity of RTI for this group of children, especially those diagnosed with low abilities, when implemented with the help of good parental support. 

#### 4.1.3. The Child–PT Bond during RTI Sessions

The RTI sessions contributed to a reoccurring sensory-motor timeframe for the children and preserved the child–PT relationship. Most therapists noticed that most children had an extreme need for movement to exercise their internal sensory self-regulation needs. The assumption was that, throughout the movement, children were more attentive in therapy and communicated better with their PT. This “feeling” noted by the PTs is supported by a growing body of evidence within the existing literature that suggests that perceptual-motor impairments are frequently present in children with ASD [44,45]. These impairments impede the modulation of sensory inputs [46,47,48,49], which can be aggravated if not discharged, as was the situation during the lockdown. Moreover, children’s postural control impairments [50] might enhance such perceptual-motor needs, leading to restlessness when internal sensory needs cannot be fulfilled due to confinement within four walls. 

### 4.2. Theme Two: The Implications of RTI on the PT 

#### 4.2.1. Preintervention Requirements for Applying RTI 

RTI required preintervention preparation of the therapeutic settings and technical tools (such as Zoom, Google Meet, and others), as suggested by others [51], along with the PT’s intention to demonstrate flexible thinking and adaptations to meet the children’s and their families’ needs. The participating PTs quickly understood that when using RTI, the therapist must be organized and pre-acquainted with the parents and child, as well as with the physical setting of the family’s accommodations. Moreover, knowing and understanding the child’s abilities and difficulties is important before implementing RTI. In the current case, most PTs were familiar to the children before lockdown, as they were their personal PTs in their kindergartens. 

RTI sessions could, therefore, be started only following a preintervention conversation with the parents, leading to an updated therapeutic contract. Such conversations aim at strengthening a therapeutic relationship and collecting information about the parents’ availability (in terms of their working status and presence of other children in the home), special needs and requests, the child’s condition, defined goals within the educational facility and their appropriateness to the home situation, the physical structure of the house, equipment and games available at home, and other important aspects. When an RTI intervention is performed within the home environment, as was the case in the current intervention, this approach helps enhance the involvement of family members, as suggested by others [51].

#### 4.2.2. The Impact of RTI on the PT’s Personal Life

Therapists were compelled to work from their own homes during the lockdown. Thus, not only did they have to adjust to the needs of the ASD children and their families, but they also had to function in an unfamiliar and complicated situation within their own families. 

Some of the PTs felt uncomfortable with the exposure of their private life. The authors believe that these feelings arose due to the combination of a lack of experience as remote therapists and the stressful situation of running one’s life and that of one’s family during a pandemic. These feelings are supported also by the findings of Kaku et al. [37], who suggested that the COVID-19 pandemic disrupted routines and impacted the coping skills of both individuals and families. We believe, however, that with better knowledge of RTI and in less stressful and more predictable times, this will not be an issue. Physical therapists must, therefore, be prepared in advance to conduct RTI, and such a training program will enhance their confidence and proficiency in using RTI. 

#### 4.2.3. The Impact of RTI on the PT’s Professional Experience

In the initial stages of RTI implementation, the PTs were concerned about their ability to indirectly engage the children’s cooperation while helping them improve their motor functioning. Similar concerns regarding physically oriented treatments were raised by others as well [36]. However, with time and experience, the PTs increasingly adjusted to the new situation, experienced fewer technological challenges, and focused on improving their services. RTI was found to have a significant beneficial impact on the PTs’ professional experience and their ability to improvise by adding innovative skills to their professional toolbox. 

The authors support the comments made by the therapists and suggest that any organization working with individuals with ASD should take this learning experience as a wake-up call. Every potential worker involved in RTI should be trained for this job in advance. This premise is supported by instructions published by the Israeli Ministry of Health [50].

#### 4.2.4. The Role of Teamwork during RTI

Physical therapists indicated that good, coordinated teamwork is essential for supporting families and therapists while providing or integrating RTI in the treatment of ASD children. The basic working habits and the quality of team members’ communication highly affected the ability to reach mutual decisions and creative solutions for ongoing challenges and dilemmas. High-quality teamwork in the ASD context has also been highlighted by others [52].

When the kindergarten staff exhibited flexibility, and the teamwork was less centralized, the PT could more easily adapt to this new and unfamiliar treatment approach. 

### 4.3. Theme Three: Therapeutic Modifications for Applying RTI

#### 4.3.1. Using RTI to Collect Information on the Child

RTI provided the opportunity for the PTs to observe the child’s spontaneous behavior in their natural environments directly. This information is essential to obtain a comprehensive picture of the child’s behavior. The pandemic offered new opportunities to overcome this knowledge gap, as was also suggested by others [51], which was quite a blind spot before, as information was mainly based on subjective parental reports. 

These findings agree with home-based and family-centered approaches, suggesting that even a brief home-based intervention is a viable and effective means of providing personalized support [38,51,53]. 

#### 4.3.2. Setting Therapeutic Goals for RTI 

The data provided by the PTs suggest that goals in the therapy room during hands-on therapeutic sessions might differ from goals set for RTI sessions. During conventional therapeutic interventions within the educational setting, therapeutic goals are constructed according to the assessment of the child and his or her parents’ objectives. With RTI, on the other hand, the initial goal may be to continue caring for and supporting the child and his or her family, especially during the initial sessions, as reported also by others [36]. The special circumstances surrounding RTI (i.e., low parental availability, the new situation, the child’s coping ability, available equipment at home, etc.) required more adaptations and flexibility, as reported by others [38], mainly in the beginning and during the adjustment period of both the child and his or her family and of the PT. 

#### 4.3.3. Therapeutic Principles of RTI 

Online RTI sessions in which the child and the therapist were able to see and communicate directly through the internet were reported to be extremely important, although only about a quarter of the children achieved such interaction. Yet, any approach, direct or indirect, must include suitable adaptations when implementing RTI. The following paragraphs will elaborate on this idea.

- Preintervention considerations and adaptations: RTI is a unique therapeutic approach that requires specific adaptations which include mastering technical skills, prearranging each therapeutic session according to the needs of the specific child, flexibility with respect to each therapeutic situation, as well as sensitivity and ability to adapt to the needs of the individual child and his or her family. These elements are echoed in an article by other researchers [38]. In order to plan RTI sessions, each family’s individual conditions must be considered (i.e., family composition, home structure, available equipment, technical possibilities, etc.). Moreover, due to differences in home vs. educational settings and conditions, we highly recommend performing a home visit prior to the RTI implementation. This will help the PT better implement the RTI in accordance with the child’s home conditions and nearby possibilities (e.g., private yard, neighborhood park, or playground).
- The role of routine in RTI: indirect interventions, repetitions, a consistent setting, and a preplanned session program were all found to be basic therapeutic principles in RTIs. These initial principles helped children with ASD participate and cooperate throughout the RTI sessions and adhere to the intervention. Here, in this unexpected situation, the known clinical versatility of children with ASD [54] was even more pronounced: although RTI sessions were a completely new modality for all of the children (as well as for the parents and the PTs), some constantly depended upon their familiar and “good old” routine (e.g., the session’s opening, a familiar setting, and a closing song), while others only needed such anchors in the initial session and then required variety in every RTI session. Naturally, daily routines and settings at the children’s homes differed from those maintained in the kindergartens, and the therapists had to cope with those differences while maintaining a therapeutic routine.
- Practical recommendations: practical therapeutic principles were studied through the RTI, suggesting some resemblance between in-person interventions and RTIs. Although the intervention model changed, the children with ASD did not. Therefore, looking at the child’s interests was the first step in building a modified relationship with him or her through RTIs. Reflecting on the dynamics of the children’s different situations, reactions, and feelings seemed to help the children communicate better with their PTs and overcome the initially confusing novel therapeutic situation. Moreover, songs, familiar music, and social stories helped capture the children’s attention and promote their cooperation. In many cases, using an agreed-upon signal, such as a hand clap or vocal call, was useful in getting their attention back. PTs specifically emphasized the crucial effect of speaking and approaching the child directly while sending video clips in order to enable the child to see the PT continuously.

#### 4.3.4. Parental Involvement

The PTs in our study repeatedly emphasized the tremendous contribution parental involvement had to the implementation of the RTIs. In fact, this observation is in line with previous studies that claim that parental involvement is an essential key to treating children with ASD [38,55,56,57]. Moreover, recent reports reinforce this consensus, especially following nationwide lockdowns, when RTIs were the only possible way to continue therapeutic work with patients, including children with ASD [36]. The unique lockdown conditions magnified the substantial role that parents played in the success of RTI sessions [38], as most children needed their parents to moderate the sessions. 

### 4.4. Theme Four: Therapist–Parent Rapport as a Necessary Basis for RTIs

Good relationships between the PTs and the parents, as well as concern for the child by the therapist and “perseverance” and “positivity” by the parents [38], encouraged parental involvement, contributing to RTI success and enhanced communication between parents and their children, creating a self-supporting triangle. The PTs also perceived that the child–parent connection was significantly strengthened when parents participated actively in the RTIs. Nevertheless, the therapists also identified distinctive differences between the parents in terms of technical and communicational abilities, availability for the therapeutic sessions, involvement, and manner of moderating the therapeutic sessions. Such differences have been reported previously, suggesting a connection between parental challenges and low adherence to different types of intervention, such as behavioral, developmental, or alternative, when parental involvement is required [58]. 

#### 4.4.1. Dynamics of the Relationship

Direct communication between PTs and parents helped build a trustful relationship and promoted parental understanding of the advantages of physical therapy for their child’s development. At the same time, it also helped the PT better understand the challenges faced by parents of children with ASD, as also reported by Wood de Wilde and associates [38], which, in turn, led to adjustments in the intervention’s goals and program accordingly. 

We speculated that during a nationwide lockdown, the parents’ stress level (regarding the family members’ financial and general health status, as well as running the family routine) would negatively influence their adherence. We found that it was necessary to schedule preintervention time with the parents to establish a new contract with them as a trustful basis for PT–parent communication and cooperation, to solve technical problems, and to gather information regarding the setting and available equipment. 

Direct communication throughout the RTI contributed significantly to its success, especially when the parents acted as partners in the process and were able to cope with challenges and overcome any obstacle. For example, sibling involvement, coordinated by parental support and mediation, enhanced the children’s cooperation and engagement. This notion that including siblings of children with autism in the interventional setting can reduce stress, as well as strengthen the entire family, was previously reported [59].

#### 4.4.2. Perceptions of the Child’s Functional Abilities 

Parents had the opportunity to understand the essence of physical therapy and the benefits it offers in terms of their child’s developmental process.

### 4.5. Limitations 

This article was based on a relatively small number of PTs (N = 13) (although those therapists treated 244 children in-person and while using RTI during the lockdown period) and, therefore, might not reflect the full scope of the situation, as more than 100 PTs were involved in this endeavor during this period.

This article describes an RTI intervention for children of ages 2 to 7 diagnosed with ASD. According to others [36], results and conclusions may differ when other age groups are involved, and, therefore, caution should be exercised when generalizing the present results to other age groups. 

The present program was implemented within the context of a national lockdown due to COVID-19, and parents were influenced by the situation; therefore, applying a similar program without the outer stressors (COVID-19, lockdown, both therapist and parents at home with children) might yield even better outcomes. 

Technological issues that were reported as barriers to therapists and parents alike are most likely to be reduced with futuristic technological advancements, thereby reducing barriers to RTI implementation [51].

### 4.6. Recommendations

At present, PTs’ ability to integrate innovative technologies (such as videocall and other platforms) into their working activities should be rehearsed and improved to better respond to the unique healthcare needs of children with ASD and their families. 

The authors support the notion that every child with ASD and his/her family should have an organized preplanned daily activity program. This idea is based on numerous previous articles, according to which such programs should be custom-adapted to each child and implemented at a younger age for increased effectiveness [60,61,62,63]. Today, after accumulating experience with RTIs, the opportunity is open to promote daily home programs by guiding and supporting the families who implement them. Such programs should be enhanced in cases where the child cannot participate in daily kindergarten activities (due to hospitalization of the child or a family member, long holidays, family relocation, etc.), thereby enabling continuity of treatment despite the break in routine. 

RTI is an emerging and promising therapy field, so PTs and other health professionals should undergo organized and professional training regarding this form of therapy, which will probably become common practice in the future. Moreover, our experience suggests that PTs should be taught remote rehabilitation as a conventional form of intervention within ongoing PT studies or as extracurricular courses.

The urgent nature of the RTI implementation due to the pandemic led to the understanding that a large, assembled, and categorized online exercise inventory will highly enhance therapists’ readiness at all times and in any case [64].

## 5. Conclusions

Remote telehealth interventions are currently emerging as a promising resource that enables the support of children with ASD and their families, as well as other pediatric clients [65], ensuring continuity of care even in extremely stressful times [36,37]. Moreover, as this relatively new concept has been implemented with relative success during a worldwide healthcare crisis, it is obvious that it will be even easier to implement in calmer times. This international crisis may, therefore, turn out to be an opportunity, as it introduced a new form of therapy for individuals with ASD and their families [36]. This notion is supported by the fact that the circumstances led to a general surge in RTI in Israel and around the world, with a 57.7% increase in the use of RTI by pediatric therapists [30]. The benefits of the RTI program appear to be highly relevant for the continuity of care, as children with ASD are particularly susceptible to a decline in their abilities and behavior when their schedule is abruptly disrupted.

## Figures and Tables

**Table 1:** Demographic data of participating PTs.

PT	Age (Years)	Gender	Experience as PT (Years)	Experience with ASD (Years)
1	39	F	9	8
2	36	F	13	2
3	51	F	25	6
4	40	F	12	7
5	60	M	32	10
6	48	F	23	10
7	37	F	14	5
8	40	F	13	1
9	41	F	17	8
10	35	F	10	4
11	34	F	8	2
12	34	F	9	6
13	38	F	11	9
Mean (SD)	41.4 (±7.6)	12F/1M	15.1 (±7.3)	5.8 (±2.9)

Abbreviation list: PT = physical therapists; ASD = autism spectrum disorder; SD = standard deviation; F = female; M = male.

**Table 2:** Organization of the PTs’ reports into themes and subthemes.

PT	Theme	Subthemes
1	The implications of RTI on children with ASD	The effect of staying at home on children’s behavior;
		2. The relation between ASD severity and the ability to receive RTI services;
		3. The child–PT bond during RTI.
2	The implications of RTI on the PT	Preintervention requirements for applying RTI;
		2. The impact of RTI on the PT’s personal life;
		3. The impact of RTI on the PT’s professional experience;
		4. The role of teamwork during RTI.
3	Therapeutic modifications for applying RTI	Using RTI to collect information on the child;
		2. Setting therapeutic goals for RTI;
		3. Therapeutic principles of RTI: Preintervention considerations and adaptations; The role of routine in RTI; Practical recommendations;
		4. Parental involvement.
4	PT–family rapport as a necessary basis for RTI	Dynamics of the relationship;
		2. Perceptions of the child’s functional abilities.

Abbreviation list: RTI = remote telehealth interventions; ASD = autism spectrum disorder; PT = physical therapist.

## Data Availability

The complete dataset containing participants’ responses divided into identified themes and subthemes is available from the corresponding author.
